# Psychoeducational Interventions for Caregivers of Persons With Multiple Sclerosis: Protocol for a Randomized Trial

**DOI:** 10.2196/30617

**Published:** 2021-08-26

**Authors:** Sara L Douglas, Matthew Plow, Tanya Packer, Amy R Lipson, Michelle J Lehman

**Affiliations:** 1 School of Nursing Case Western Reserve University Cleveland, OH United States; 2 School of Occupational Therapy Dalhousie University Halifax, NS Canada; 3 School of Health Administration Dalhousie University Halifax, NS Canada

**Keywords:** multiple sclerosis, caregivers, distress, anxiety, depression, psycho-education, website, coaching, mobile phone

## Abstract

**Background:**

Of the approximately 1 million people living with multiple sclerosis in the United States, more than half receive informal, unpaid care or support from family or friends (caregivers). These caregivers report high levels of stress, anxiety, and negative emotions. Few researchers have conducted psychoeducational interventions for these caregivers.

**Objective:**

This paper presents a protocol for a randomized clinical trial that aims to test the efficacy of two interventions for improving stress, anxiety, depression, and negative emotions for caregivers of persons with multiple sclerosis.

**Methods:**

Participants included any self-identified family or friend caregiver of a person with multiple sclerosis. Data collection began in April 2021 and is expected to continue until November 2021. Participants will be randomized to receive either a website-only or a website-coaching intervention delivered for 6 weeks. Data will be collected at baseline, 6 weeks after baseline (after delivery of intervention), and 6 weeks later.

**Results:**

The protocol was approved by the institutional review board of the Case Western Reserve University on January 21, 2021 (protocol 20201484). As of May 2021, 66 participants were enrolled.

**Conclusions:**

Our findings will have implications for identifying the efficacy of two types of interventions developed for caregivers of persons with multiple sclerosis to reduce negative psychological outcomes associated with caregiving.

**Trial Registration:**

ClinicalTrials.gov NCT04662008; http://clinicaltrials.gov/ct2/show/NCT04662008

**International Registered Report Identifier (IRRID):**

DERR1-10.2196/30617

## Introduction

### Background

Currently, there are approximately 1 million people with multiple sclerosis in the United States [1], with an estimated 46%-58% of them receiving informal, unpaid care from family or friends in the course of their illness [2,3]. These caregivers are critical in maintaining high-quality care and support for persons with multiple sclerosis and often provide care not routinely provided through the established health care system [4].

These informal caregivers, such as those of persons with other chronic illnesses (eg, Alzheimer disease and cancer), provide a variety of care and support that often vary according to the needs of the person with multiple sclerosis [5,6]. They are often frustrated by a lack of information regarding how to accommodate the changing needs of the person with multiple sclerosis, deal with the uncertainty of the course of the illness, and find support for their own emotional and physical needs [5,7-9]. Research has found that these caregivers experience negative physical and psychological outcomes that tend to increase as the disease progresses and care needs increase [10-13]. For example, these caregivers have a lower health-related quality of life [14,15], elevated levels of fatigue [16], and significantly higher levels of anxiety (68%), depression (44%), and distress (51%) [5,17-19]. In addition, these caregivers have reported that their increased anxiety, distress, and burden not only worsened their health but also had a negative impact on their employment. Approximately 24% of caregivers reported that they had to reduce or stop working because of the demands of caring for a person with multiple sclerosis [3], thus placing an additional source of stress on this caregiver group. In addition, these caregivers report concerns about the possibility of relapse or progression of the disease—concerns that lead to anticipatory grief for the future [6,20]. Thus, they must not only deal with the current care and emotional needs of the person with multiple sclerosis but also often deal with their own grief about the losses that potentially lie ahead.

Research examining the needs of caregivers of persons with multiple sclerosis has highlighted the need to reduce caregiver distress and sense of burden [5,17,21,22] (which often precede caregiver anxiety and depression), provide information relevant to caring for a person with multiple sclerosis [5,23], and provide information and skills to facilitate communication [24,25]. Strategies that include psychoeducational programs providing information and support have shown promise [5,9,20]. Interventions supporting caregivers of persons with other chronic illnesses (eg, cancer) have shown that the use of tailored strategies to increase caregiver self-efficacy and provide emotional support has effectively reduced distress, anxiety, and depression [26-31].

Although caregivers of persons with multiple sclerosis report poor emotional and physical outcomes, to date, few researchers have tested interventions for these caregivers. Interventions delivered to both persons with multiple sclerosis and their caregivers [24,25,32] and interventions delivered solely to caregivers [23,33-35] have been published. Of the interventions focused solely on caregivers, the types of interventions varied, including a group psychoeducational intervention [23], a psychoeducational intervention for empowerment [33], a cognitive behavioral web-based mindfulness intervention [34], and a behavioral intervention providing information and skills related to patient mobility problems [35]. Outcomes have focused on caregiver burden, empowerment, anxiety, and depression—factors shown to relate to ongoing emotional and physical dysfunction [9,23-36]. Only one of the caregiver-focused intervention used an individualized psychoeducational intervention—despite the success of this type of intervention in other caregiver populations [36-39].

Psychoeducational interventions, as conceptualized for this study, encompass a broad range of activities that combine education and supportive activities (eg, counseling and support to adopt self-management strategies). These interventions are tailored to individuals and can be delivered individually or in groups [39,40]. Some psychoeducational studies have reported effectiveness in improving psychological outcomes [31,37,41,42] and self-efficacy for care tasks [43]. In addition, some studies using coaching reported that this approach is effective in providing individualized information and emotional support [44]. The strategy of providing individualized coaching sessions for this study was based on research indicating that psychoeducational interventions, which are multidimensional, individualized, and flexible, are most effective [37].

The use of websites and other technologies (eg, videoconference) to deliver interventions has been well established, feasible, and acceptable to patients and caregivers [40,45-49]. In addition, previous research has noted the benefits of eHealth interventions for anxiety and depression, support for self-management activities, and improvement of family functioning in a variety of populations [41,43,46,48]. The use of videoconference and telephone to deliver coaching sessions to caregivers in our previous study [31,49] was well-received by caregivers.

The conceptual model underlying this study intervention is the Stress Appraisal Model, which identifies caregiving as a dynamic process involving caregivers and care receivers. The key to this model is the idea that stress influences the appraisal of caregiving, which, in turn, influences the caregiver’s psychological response [50]. Thus, stress is a proximal outcome of perceived burden and poor psychological outcomes. Testing the model using path analysis has demonstrated that delivering emotional support and information (as in a psychoeducational intervention) enhances the caregiver’s sense of self-efficacy and reduces the appraisal of caregiving, which improves psychological outcomes [50].

### Objectives

To date, caregivers of persons with multiple sclerosis remain an understudied group of caregivers who demonstrate poor psychological outcomes throughout the trajectory of their loved ones’ illness. These negative psychosocial outcomes can have significant impacts on caregivers’ physical health and their abilities to support and provide quality care to their loved ones (the patient) [19,51]. A tailored psychoeducational intervention can alleviate some of this stress [40] by providing web-based information—available 24 hours per day, 7 days per week to address questions and concerns of caregivers of persons with multiple sclerosis. In addition, individualized coaching from trained professionals familiar with the needs of this caregiver group may enhance their sense of self-efficacy as a caregiver and decrease distress, anxiety, depression, and negative emotions [5,21,44,52,53].

This randomized clinical pilot trial aims to compare the effectiveness of a tailored website-coaching intervention (delivered via videoconference or telephone) with a website-only intervention on caregivers’ negative emotional state, distress, anxiety, and depression. Changes in outcomes between the intervention groups will be compared over time (baseline, 6 weeks, and 12 weeks). We hypothesize that the website-coaching intervention will yield a greater reduction in negative emotions, distress, anxiety, and depression over time compared with the website-only group.

## Methods

### Setting

This study takes place virtually using a website designed for the study (delivered to both groups) and coaching via videoconference or telephone (website-coaching group only) to adult caregivers of persons with multiple sclerosis who have responded to recruitment outreach throughout the United States. Trained research assistants will obtain informed consent and collect data remotely using REDCap (Research Electronic Data Capture). REDCap is a secure web application for building and managing web-based surveys and databases for research studies [54].

### Participants

We used two convenience sampling strategies to recruit the study participants. One strategy uses targeted recruitment using a list of persons with multiple sclerosis, as indicated in a previous study (Patient-Centered Outcomes Research Institute [PCORI] grant Multiple Sclerosis-1610-37015) who are willing to participate in future research. Research assistants refer to persons with multiple sclerosis from previous studies and ask if they have a caregiver who might be interested in the study. If the person with multiple sclerosis says “yes,” then the research assistant asks for telephone contact information for the caregiver and permission to contact the caregiver. After contacting the caregiver, the research assistant screens for eligibility, explains the study (if eligible), and, if the caregiver agrees to participate, sends the electronic consent via REDCap for web-based signature.

Participants may also enter the study by responding to an email sent by the National Multiple Sclerosis Society to all caregivers in their database or by responding to a Facebook advertisement that describes the study. The email and Facebook advertisement approaches include a flier developed by the Case Western University Marketing Department and approved by the institutional review board. In both the email and Facebook approaches, potential caregiver participants use a link from the posted flier that leads them to a REDCap survey form where they provide contact information and verify that they are interested in participating in the study. The research assistants then follow the same screening and consent procedures used in the first recruitment approach.

This study aims to enroll 150 caregivers of persons with multiple sclerosis. A caregiver is defined as someone (family or friend) who provides any type of support (eg, physical, emotional, or administrative support, such as paying bills) to the person with multiple sclerosis, who is not a professional caregiver, and is not paid for their efforts [31,55]. Inclusion criteria for caregivers were (1) self-identifying as an adult (18 years or older) caregiver for a person with multiple sclerosis; (2) being capable of providing informed consent; (3) identifying English as their primary language; and (4) being able to access the internet. Exclusion criteria were (1) being younger than 18 years; (2) receiving payment for caregiving responsibilities; (3) being unable to provide informed consent; (4) not identifying English as their primary language; and (5) not having access to the internet.

### Study Procedures

Caregivers received an email that included a link to the baseline REDCap survey with all study tools after providing signed consent. Caregivers were randomly assigned to one of the two study arms after completing the baseline survey. A minimization stratified randomization technique (MinimRan) was used to balance preidentified stratifying covariates across treatment assignments [56,57]. In this study, the stratification variable was gender. The project director, who is blind to other characteristics, performs randomization and uses a computer-generated list of random numbers. This list is stored in a separate document that is unavailable to the research assistants who enroll participants.

After randomization, the project director emails or calls the caregiver with their group assignment, website link, unique password to the study designed website, and instructions regarding the name of the interventionist who will be contacting them to set up the first coaching session (for those randomized to the website-coaching group). The project director asks participants to be randomized to the website-coaching group if they will be charged minutes if they use their cell phones. To date, no one has indicated that using the telephone (should they choose) for coaching sessions will result in charges. A study cellphone will be mailed to any individual who states that they will incur individual charges for the use of their cell phone to participate in the study. All research assistants were blinded to group assignment, and all contact information for potential and enrolled participants were housed within REDCap to protect confidentiality before, during, and after the study.

The intervention began immediately after randomization and continued for 6 weeks. research assistants send emails to all participants at the end of the intervention (using REDCap) and 12 weeks after randomization to obtain all study measures. Plans to promote participant retention and complete follow-up involve providing all participants with a US $20 gift card after completion of each data collection time point. research assistants send an email to participants who do not complete their web data surveys within a week of receiving the REDCap link with a reminder message to complete the survey as soon as possible. This procedure has been successful in other longitudinal studies involving REDCap data collection [31].

### Interventions

The website was developed for this study by a website team at Dalhousie University. It is hosted on a secure server housed in Canada. The website meets all privacy requirements outlined in both the US Health Insurance Portability and Accountability Act (HIPAA) and the Canadian Personal Information Protection and Electronic Document Act [58,59]. IP addresses are collected to increase security; however, they are immediately scrambled, and neither the web team nor the research team has access to IP addresses.

No personal information is collected or stored on the website. A secure, individual password to access the website is provided by the project director to the participants when they are enrolled. The key to the list of passwords is saved in a password-protected file accessed by the project director. Participants do not interact or communicate with the research or website teams at Dalhousie.

The development of the website followed strategies used by other psychoeducational websites [40,51,53,60]. The website content and delivery was informed by (1) a systematic review of psychoeducational interventions for caregivers of persons with multiple sclerosis [M Plow, personal communication, June 16, 2021]; (2) findings from tested web-based psychoeducational interventions [31,41,43]; (3) findings from interviews and focus groups conducted with caregivers of persons with multiple sclerosis, professionals with experience working (and conducting research) with persons with multiple sclerosis and their caregivers; (4) discussions with experts in the development of educational websites (Dalhousie) and caregiver experts; and (5) use of best practices for written patient education materials (eg, avoiding jargon, defining new or complicated terms, using active voice as much as possible, breaking information into chunks, and using short sentences) [61,62].

Caregivers of persons with multiple sclerosis and persons with multiple sclerosis both provided input into the development of the website through two web-based meetings hosted by one of the study investigators (MP). Both groups provided input into the research design, the utility of intervention comparisons, recruitment strategies, and the selection of meaningful outcomes. They confirmed the overall need for the research and the importance of supporting caregivers (called *care partners* in study materials per the request of the caregivers).

The website materials consist of text information, weblinks, and video scenarios designed specifically for caregivers of persons with multiple sclerosis in the following areas: (1) information about multiple sclerosis, (2) obtaining reliable information about multiple sclerosis on the web, (3) caring for your loved one with multiple sclerosis, (4) COVID-19 and multiple sclerosis, (5) caring for yourself, and (6) planning and decision-making. The main landing pages for the website were based on findings from previous psychoeducational websites and descriptions of key informational topics provided in web-based psychoeducational interventions [41,42,63]. Investigators for the project who had expertise in the areas of caregiving (SLD and ARL), multiple sclerosis (MP, TP, and MJL), and website interventions (TP and MJL) developed the content for the project. The development of the video vignettes was based on previous research that identified the unique needs of caregivers of persons with multiple sclerosis [5,7-10,21,22], general caregiving research [26-31], and answers to a series of questions emailed to caregivers of persons with multiple sclerosis. The website developers oversaw the development and testing of the video vignettes and the overall website.

Videos were designed to support behavior change through peer modeling and social persuasion, which are two key mechanisms to support behavioral change [64,65]. They showcase caregivers discussing and modeling topics that may be of importance to caregivers in general. Videos were designed using input from caregiver members of two group meetings held with caregivers and persons with multiple sclerosis. At that meeting, caregivers responded to questions focused on each of the intervention coaching sessions. Their responses were used to construct videos reflecting their concerns and responses to concerns. Quotes from persons with multiple sclerosis (present at the meeting) were also included to provide descriptions of lived experiences. Each video vignette scenario includes a caregiver speaking to either another caregiver or friend about issues and decisions. Storyboards and scripts for video animation and voiceovers were created and refined by the research team. Once voiceovers and storyboards were complete, the videos were animated.

COVID-19 information was included in the content of the interventions (and analyses) to describe the concerns that caregivers of persons with multiple sclerosis and persons with multiple sclerosis have regarding COVID-19. Persons with multiple sclerosis are at increased risk for severe illness from COVID-19 and their caregivers want to ensure that they are doing what is right when providing care. Rather than ignoring issues around providing care that will inevitably emerge as the pandemic evolves, we have decided to directly address it to ensure that interventionists are consistent in their responses to questions and in providing information and resources. Finally, the psychological toll of the pandemic has been described for both patients and caregivers, and it is important to examine its influence on our primary outcomes of mental health [66].

**Figure 1:**
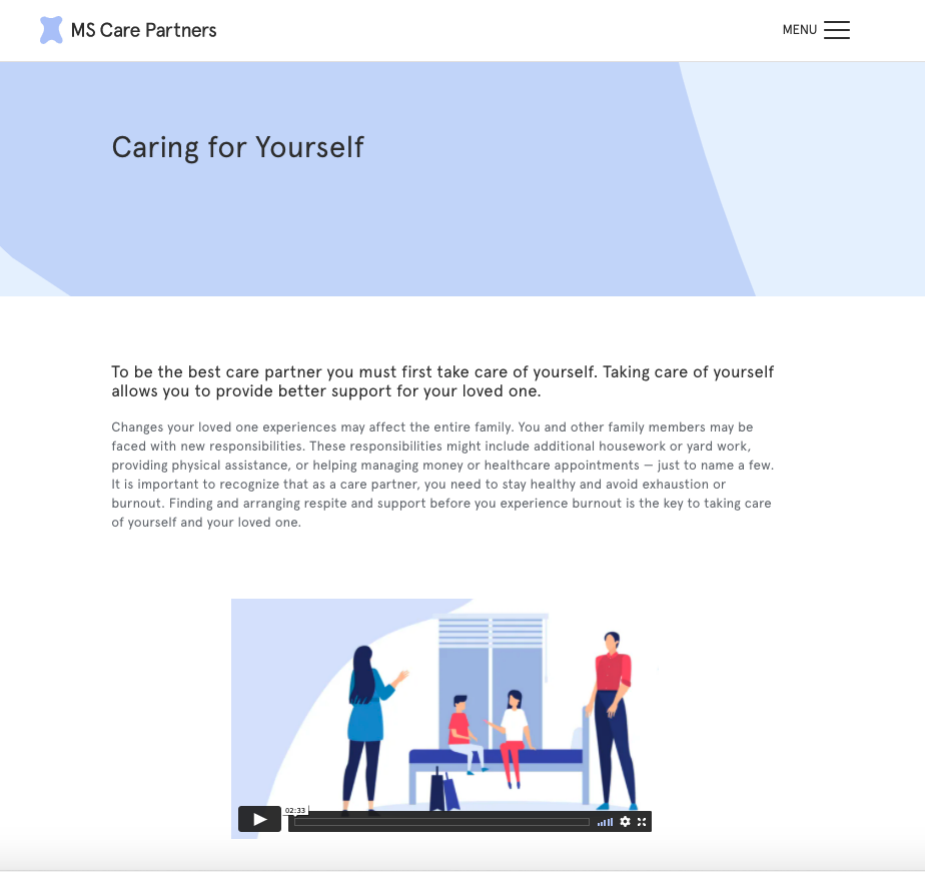
Homepage for the “caring for yourself” module from the study website.

Each key area (eg, *caring for yourself* and *COVID-19 and multiple sclerosis*) follows the same format. A brief summary providing an overview of the key area is found on the landing page (Figure 1). Participants then chose to watch a short video or find answers and external links to a series of commonly asked questions.

**Figure 2:**
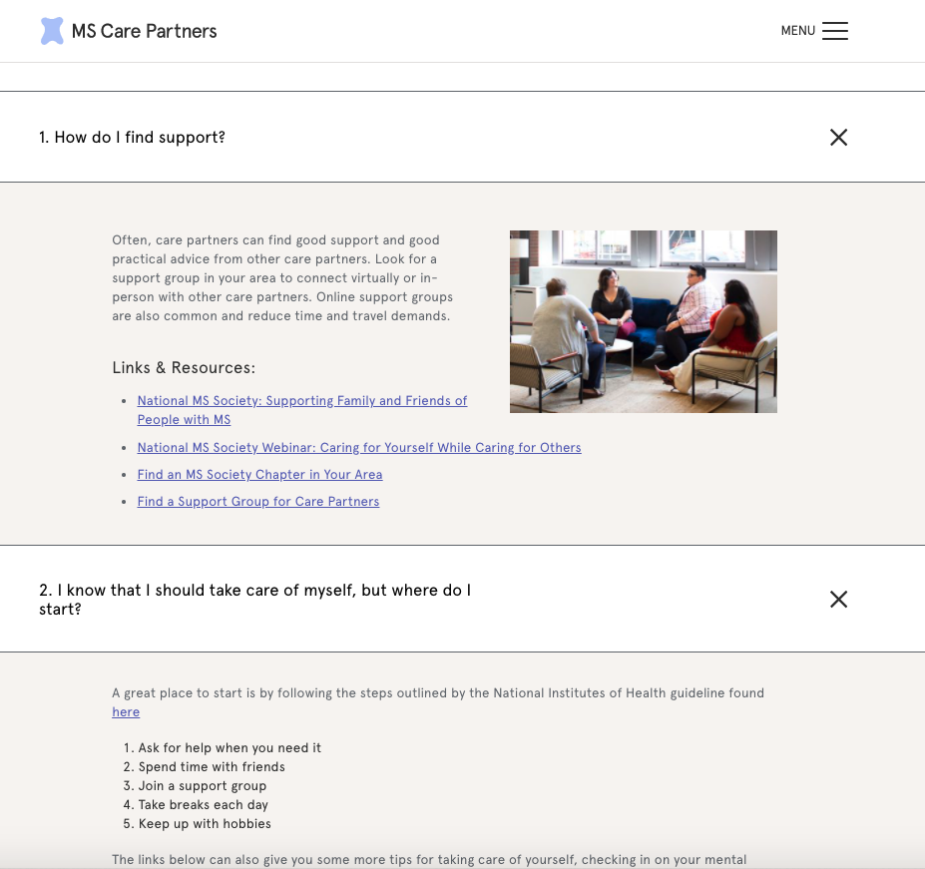
Frequently asked questions for the “caring for yourself” module from the study website.

Each video vignette (2 to 3 minutes in length) depicts a conversation between a caregiver of a person with multiple sclerosis and another individual (eg, friend, caregiver, or person with multiple sclerosis) discussing an issue identified as relevant during the caregiver engagement process before developing the website. For example, on the website page *caring for yourself*, a caregiver talks to a friend about problems finding time for himself; the other caregiver suggests strategies for dealing with this problem. The list of commonly asked questions provides the selected resources and links related to each question (Figure 2). Each participant in the study can access the website as many or as few times as they wish. At the completion of the 6-week intervention period, researchers at Dalhousie will track the frequency of website use and the most frequently visited components of the website to provide aggregate descriptive information about website use.

Participants randomized to the website-coaching group receive (in addition to access to the website) a dose of four personalized coaching sessions (for 6 weeks) from a trained interventionist (licensed independent social worker) who conducts the sessions via videoconference or telephone per each participant’s preference. Completion of these four coaching sessions constitutes a full *dose* of coaching [37]. The four key components of the coaching sessions are (1) session 1—identifying caregivers’ needs for information and support; (2) session 2—strategies for caring for a loved one with multiple sclerosis; (3) session 3—caring for yourself; and (4) session 4—planning and decision-making for the future. The key topics associated with each session are listed in Textbox 1.

**Key components of each coaching session.**

**Components of the Session**

- Session 1
  Assess caregivers’ emotional and physical distress, key needs, and needs for information and support.
  Work with caregivers to develop strategies (plan) for their most immediate need.
  Review the study website and focus on information and sources of support that are most relevant to the caregivers’ immediate needs.
  Establish goals for caregivers to focus on for next session.
- Session 2
  Review previous issues of concern and assess their status; determine if there are new needs or concerns (performed at the beginning of each session).
  Assess caregivers’ need in information or support regarding how to assist persons with multiple sclerosis with their physical needs and symptoms. Provide information, support, and resources as needed.
  Assess caregivers’ needs related to assisting person with multiple sclerosis with emotional needs or emotional symptoms. Provide information, support, and resources as needed.
  Assess caregivers’ needs related to communication with person with multiple sclerosis and family. Work with caregiver to develop strategies to assist identified communication issues.
  Provide information regarding COVID-19 and multiple sclerosis (testing, multiple sclerosis society and Centers for Disease Control and Prevention recommendations, and vaccinations).
  Discuss goals and plans for next session.
- Session 3
  Review previous issues (session 2 point 1).
  Assess caregivers’ needs related to emotional support. Provide information, support, and resources as needed.
  Assess caregiver involvement in self-care activities (eg, stress management and respite care). Provide information, support and resources as needed. Develop specific strategies to enhance identified issues regarding self-care activities.
  Discuss goals and plans for next session.
- Session 4
  Review previous issues (session 2 point 1).
  Discuss strategies for preparing for future health care provider visits (eg, questions to ask, how to get concerns addressed, and how to be an advocate for the person with multiple sclerosis).
  Discuss strategies to start planning for changing care needs of the person with multiple sclerosis (eg, evaluating whether home care services are needed and how to find assistive devices).
  Provide information and discuss strategies for making decisions and plans for advanced stages of multiple sclerosis.
  Assess, provide information on, and discuss advance care planning and palliative care.

Interventionists were trained by the project director (a social worker). First, all content and website links found in the coaching manual were reviewed. Next, the project director demonstrated a coaching session with an *actor* caregiver with interventionists then practicing each coaching session. Throughout the practice sessions, the project director provided feedback, and retraining occurred when components of the coaching needed clarity or additional practice as deemed by the project director. Finally, the project director listened to the initial coaching sessions provided to caregivers of persons with multiple sclerosis to establish baseline fidelity and consistency of the coaching delivery.

At the beginning of session 1, the interventionist conducted a brief assessment interview that focused on the caregiver’s assessment of their distress related to illness and care of the person with multiple sclerosis, their emotional and physical health, and their level of satisfaction with communication (persons with multiple sclerosis, family regarding the care of the person with multiple sclerosis, and multiple sclerosis health care providers). Finally, the interventionist asked the caregiver to identify one or two specific issues of concern regarding their role in assisting the person with multiple sclerosis. The development of this assessment was based on a review of previous psychoeducational coaching interventions [44,63] and findings from previous work providing coaching sessions to caregivers of persons with a chronic illness (cancer) [31]. The purpose of this assessment was to ensure that the intervention targeted key problems and concerns identified by the caregiver.

Each session uses standardized content but is tailored to focus on issues of relevance to the caregiver. Interventionists follow detailed outlines in the intervention manuals (with links to additional information) in providing skill-building exercises (communication, stress reduction), information, and other topics (evaluating content on the web for accuracy). Outlines were modified as needed by the interventionists based on the caregivers’ assessments. For example, a caregiver who has experience with stress reduction techniques and does not identify high distress will not receive as much depth and detail regarding stress reduction as a caregiver who has little experience with these techniques. Each session takes approximately 35 to 40 minutes based on data from current coaching sessions. The total dose for all four sessions will be 120 to 160 minutes—a dose that reduced caregiver distress and anxiety in previous work [31].

Each participant determined whether their coaching sessions were delivered via telephone or videoconference. For those selecting videoconferences, Zoom (Zoom Technologies, Inc) is the app used for their sessions. We chose this app based on the recommendation of the Case Western Reserve University’s Information Technology department because it provides end-to-end encryption, and we can prohibit invitees from recording videoconference sessions [67]. Caregivers can join Zoom coaching sessions on smartphones, tablets, laptops, or desktop computers.

### Outcomes

The primary outcome was caregiver distress, and the secondary outcomes were anxiety, depression, and negative emotions. Distress is the primary outcome, as distress contributes to the development of anxiety and other negative emotions [68]. All outcome measures were caregiver-reported outcomes assessed using psychometrically sound tools and were analyzed using the metric of change from baseline.

### Sample Size

The sample size needed to address the major aim of the study (N=150) was calculated using the Hedeker formula [69] for a repeated measures mixed effects model and included the following assumptions: a power of 0.80, correlation among 3 repeated measures of 0.5, a small Cohen *d* effect size of 0.35, and an attrition rate of 20%. These assumptions are consistent with previous studies on unpaid caregiver interventions reporting clinically meaningful changes in mental health outcomes [31,37,41,42,70].

Given that this is a pilot study, the analyses are considered exploratory in nature. Given the absence of studies testing interventions for caregivers of persons with multiple sclerosis, we will identify potential moderators a priori before testing them in the full analyses and will not test them simultaneously. As a result, we will have adequate power to detect medium to large effects using a structural equation modeling approach for our full analyses.

### Study Instruments

All tools were selected based on psychometric properties, clinical applicability, and low participant burden. When possible, tools from the National Institute of Health Toolkit were used.

The *overall negative emotional state* was measured using the Depression Anxiety Stress Scale-42 [71]. This instrument consists of 42 statements representing negative emotional states of depression, anxiety, or stress (eg, “I found it hard to wind down” and “I felt sad and depressed”) during a 7-day recall period. The Depression Anxiety Stress Scale-42 uses a 4-point Likert scale ranging from 0 to 3, with higher scores representing a greater amount of time experiencing each statement of negative emotion (eg, 3=applied to me most of the time). Three subscale scores (depression, anxiety, and stress) were computed, and all items were summed to compute a total composite negative emotion state score. The total composite score will be used for analytic purposes, with higher scores representing a greater amount of time associated with overall negative emotions. The tool has excellent reliability and validity, with Cronbach α ranging from .88 to .94 [71], indicating that the items are homogeneous and measure a single construct.

*Anxiety* and *depression* were measured using the short-version Patient-Reported Outcomes Measurement Information System (PROMIS) instruments PROMIS-Anxiety and PROMIS-Depression, each of which has excellent psychometric properties [72]. The instruments consist of four items, use a 7-day recall period, and use a 5-point Likert scale ranging from 1 to 5, with higher scores representing more of the domain (anxiety or depression). Raw scores are converted to standardized T-scores for analysis following the PROMIS guidelines (mean 50, SD 10). Higher scores indicate greater levels of either anxiety or depression. Cutoff scores that classify scores as normal, mild, moderate, or severe are validated [70,73].

*Distress* was measured using the National Comprehensive Cancer Network distress thermometer tool. This tool is a single-item, self-report measure of psychological distress and has excellent psychometric properties for caregivers [74]. Participants rated their distress in the past 7 days using an 11-point visual analog scale ranging from 0 (no distress) to 10 (extreme distress), with higher scores indicating higher levels of distress. Scores ≥4 represent clinically elevated levels of distress for caregivers [75,76]. The use of the distress thermometer will allow us to examine its applicability within the caregiver of persons with multiple sclerosis population and compare our caregiver sample and other caregiver groups.

### Data Collection

At study enrollment, a web-based survey within REDCap was used to obtain demographic information and baseline outcome measures (negative emotions, anxiety, depression, and distress). The same measures and procedures are used 6 weeks later (the end of the intervention period) and once more 6 weeks later (to assess ongoing effects). Caregivers in both study arms answered the same survey questions. We found that participants completed the measures in approximately 8 to 10 minutes.

For participants receiving coaching sessions, our interventionists tracked the time spent in coaching visits with each caregiver so that we could describe the time required for delivery of that component of the intervention. Website use is tracked via participants’ unique password log-ins to describe the frequency of use, commonly used portions of the website, and patterns of use for participants in both arms of the intervention. The data are presented in the aggregate.

### Fidelity of the Intervention

Throughout the study period, we will randomly select 15 participants randomized to receive coaching sessions. All coaching sessions are observed by an independent expert (with permission from the caregiver). The expert evaluates whether the core topics for each session are presented by the interventionists as outlined in the intervention manual using a checklist (Multimedia Appendix 1). Afterward, the project director analyzes the consistency between the expert and interventionists’ notes (housed in REDCap) using checklists for each session and following a protocol established in previous work [31]. This procedure ensures the robustness and reproducibility of the intervention. Retraining will occur if the agreement between the interventionist and the independent expert falls below 80% (12/15) of the comparisons—to date, our cumulative agreement rate is 100% (5/5). We have successfully used this approach in other caregiver intervention studies [31,77].

### Safety Monitoring and Adverse Events

A data and safety monitoring plan was established for the study. On a quarterly basis during the enrollment and data collection period, our research team will review data regarding recruitment, refusals, attrition, differential (study group) attrition, morbidity, and mortality.

Owing to the potential clinical significance of the anxiety and depression data, we will monitor potential anxiety and depression through an evaluation of participants’ T-scores on both the PROMIS-Depression short form 4a and PROMIS-Anxiety short form 4a tools. If a participant has a depression score ≥75.7 (depression) or an anxiety score ≥77.9 (these scores represent the upper 10% in a range of scores), the research assistant will contact the participant via telephone within 48 hours of receiving the score. The research assistant will recommend the participant contact their primary care provider for further evaluation and, in addition, will provide links to the American Psychological Association and State Psychology Associate Therapist databases where they can find names of mental health professionals in their geographic location. This reaction management protocol has been used previously in a large randomized controlled trial that tested this intervention in a cancer caregiver population [31]. All adverse events, concerns, or problems identified by the research team will be reported to the institutional review board and then to the funding agency.

### Data Management

All data will be collected via REDCap and downloaded for use with SPSS and SAS statistical packages. Data management and cleaning involve frequencies for range checks for data values for all variables. Missing data will be examined and imputed as outlined in the PROMIS scoring manual for PROMIS measures. The distress thermometer uses a single item; therefore, cases with missing data for this item will not be included in analyses involving distress. Missing data for other tools will be imputed using mean imputation at the tool level.

### Statistical Methods

Data will be analyzed using SPSS and SAS, and all investigators, along with the statistician and data manager, will have access to the final data set. Before conducting multivariate analyses to examine group differences, we will use descriptive techniques to examine univariate characteristics and bivariate relationships among variables, covariates, and outcomes. These techniques will be based on proportions, medians, and means. We will also describe (for each variable) frequencies and measures of central tendency for all variables and assess data for violations of assumptions for all planned statistical tests. Data transformations will be used to remedy issues concerning nonlinearity or high skewness.

To address the primary outcome (distress), a linear mixed effects model will be used to test whether there is a significant difference between intervention groups in time on the caregiver outcome of distress. The linear mixed effects model will include the variables of group assignment, time, and the interaction of time by group, along with the participant-specific random intercept and slope. Intention-to-treat and per-protocol analyses will be conducted. The linear mixed effects model should be sufficient in most cases to account for missing data in the intention-to-treat analysis. Participants who provide complete data and attend three or more coaching sessions will be included in the per-protocol analysis. We will also explore the influence of COVID-19 anxiety and website usage as covariates and test for moderation by multiple sclerosis disability, race, ethnicity, rural versus urban location of the caregiver, caregiver COVID-19 positive or negative status, whether the caregiver is local or distant from the person with multiple sclerosis, and socioeconomic status. The analytic approach for the secondary outcomes (anxiety, depression, and negative emotions) will be identical to that used for examining the primary outcome of the study.

The study results will be communicated to the participants via email and publication in patient-focused venues, such as the National Multiple Sclerosis Society’s website [78]. In addition, the results will be reported to the sponsor in the final report and via publications and presentations to health care providers in appropriate journals (eg, the *Multiple Sclerosis Journal* and the *Journal of Neurological Sciences*) and conferences.

## Results

This study was funded in November 2020 by the PCORI. The research protocol was approved by the institutional review board committees of the Case Western Reserve University (January 21, 2021, protocol 20201484) and Dalhousie University (March 23, 2021, protocol 20215484). The study was registered with ClinicalTrials.gov (NCT04662008). Data collection began on April 1, 2021, and as of May 2021, we enrolled 66 participants.

## Discussion

### Principal Findings

There are two main areas of innovation related to this study that add to science. First, caregivers of persons with multiple sclerosis have been the sole focus in only four intervention studies (one was a pilot study), despite the documentation of significant and ongoing needs for strategies to reduce their poor psychological outcomes [5]. This is the first study to examine the effects of a tailored psychoeducational intervention on this vulnerable group of caregivers and will add to the science of caregiving and the evolving science of caregiving of the person with multiple sclerosis.

In addition, this study will test two types of interventions for caregivers of persons with multiple sclerosis. The website-only group intervention represents the delivery of information and support that is self-directed as the person chooses what information is of interest and relevance. However, the website-coaching group enables information and emotional support to be tailored to the caregiver’s needs and allows for professional guidance for skill-building, understanding information, and receipt of emotional support. In addition, although our sample size is not large enough to incorporate many covariates, we will be able to examine the impact of a few caregiver variables (eg, gender, race, and hours of caregiving provided) on the efficacy of the two interventions. This information will guide the refinement of interventions for future testing in a larger study of caregivers of persons with multiple sclerosis.

By examining patterns of use of the website, we will also be able to add to the understanding of the issues and concerns that are of most interest to caregivers of persons with multiple sclerosis. Although previous research has provided some descriptive data [6,8,10], these studies have primarily focused on describing poor psychological outcomes [4,5,14,15], coping strategies [7,9,13], or stressors [6,18,20], identified by caregivers of persons with multiple sclerosis. We will be able to identify topics of most interest (eg, self-care activities) and questions of greatest concern (eg, “How is multiple sclerosis treated?”) elicited directly from website usage of caregivers of persons with multiple sclerosis. This will provide new information and guide recommendations for refining both interventions in preparation for large-scale testing. Similarly, by evaluating aggregated comments from interventionists’ coaching sessions, we will be able to assess issues of greatest concern and topics of greatest interest for caregivers at different points in the trajectory of the illness.

### Conclusions

There remains a lack of data regarding strategies to assist caregivers of persons with multiple sclerosis at different points along the course of illness. Results from this pilot study will determine whether either or both of these interventions provide clinically meaningful improvements in caregivers who are providing care at different points along the caregiving trajectory. Data from this study will provide insights regarding issues of concern for this group of caregivers and guide the refinement and large-scale testing of interventions for this group of vulnerable caregivers.

## Appendix

Multimedia Appendix 1 Fidelity checklist example.

## Abbreviations

HIPAA: Health Insurance Portability and Accountability Act
PCORI: Patient-Centered Outcomes Research Institute
PROMIS: Patient-Reported Outcomes Measurement Information System
REDCap: Research Electronic Data Capture
