# Supplementary material for: Psychoeducational Interventions for Caregivers of Persons With Multiple Sclerosis: Protocol for a Randomized Trial
Source: JMIR Res Protoc. 2021 Aug 26;10(8):e30617. doi: 10.2196/30617 (PMC8430872; doi:10.2196/30617)
Supplement: Multimedia Appendix 1 [file resprot_v10i8e30617_app1.pdf]

SESSION II: Caring for a Loved One with Multiple Sclerosis

No	Suggested Questions/Prompts	Check (if discussed)
1. Touching Base	• Review any additional information obtained from the initial assessment.<br>• Review concerns/plan from last session and assess current issues/needs/patient status with Caregiver (CG) (review concerns/plan from last session)<br>• Answer any questions that have arisen since your last meeting.<br>• Review any "homework" that the CG was to have done in preparation for this session.	
Notes re. touching base		
2. Assess Needs Related to Assisting Person with MS with Physical Needs or Symptoms<br><br>Provide information and support	• Ask CG if their loved one with MS is experiencing any physical symptoms associated with MS: fatigue, numbness, blurred vision, bowel/bladder dysfunction, walking difficulties, disrupted sleep, weakness, dizziness.	
	• Show CG study website and show them the National MS Society Brochure related to common symptoms of MS and the Guide for Caregivers.<br>• Review on study website and MS Society brochure information directly related to symptoms identified above by CG.	
	• If additional information is needed, go to specific websites on the study website such as (www.cidrr8.research.va.gov/rescue/physical-needs/personal-care.cfm#tips) listed there. Guide them to the appropriate information and discuss whether this is would be a helpful strategy.	
	• Help CG develop a specific strategy for assisting their loved one with one symptom of high concern. Outline steps that the CG can use to apply this approach for other symptoms of concern.	
	• If additional information is needed regarding how to assist physical care needs, include this as a goal at the end of this session and identify sources of information that you can share with the CG at the next session.	
